# Multispectral imaging reveals the tissue distribution of tetraspanins in human lymphoid organs

**DOI:** 10.1007/s00418-015-1326-2

**Published:** 2015-05-08

**Authors:** Charlotte M. de Winde, Malou Zuidscherwoude, Angela Vasaturo, Alie van der Schaaf, Carl G. Figdor, Annemiek B. van Spriel

**Affiliations:** Department of Tumor Immunology, Radboud Institute for Molecular Life Sciences, Radboud University Medical Center, Geert Grooteplein-Zuid 26, 6525 GA Nijmegen, The Netherlands

**Keywords:** Multispectral imaging, Tetraspanin, CD37, CD53, Immune system

## Abstract

Multispectral imaging is a novel microscopy technique that combines imaging with spectroscopy to obtain both quantitative expression data and tissue distribution of different cellular markers. Tetraspanins CD37 and CD53 are four-transmembrane proteins involved in cellular and humoral immune responses. However, comprehensive immunohistochemical analyses of CD37 and CD53 in human lymphoid organs have not been performed so far. We investigated CD37 and CD53 protein expression on primary human immune cell subsets in blood and in primary and secondary lymphoid organs. Both tetraspanins were prominently expressed on antigen-presenting cells, with highest expression of CD37 on B lymphocytes. Analysis of subcellular distribution showed presence of both tetraspanins on the plasma membrane and on endosomes. In addition, CD53 was also present on lysosomes. Quantitative analysis of expression and localization of CD37 and CD53 on lymphocytes within lymphoid tissues by multispectral imaging revealed high expression of both tetraspanins on CD20^+^ cells in B cell follicles in human spleen and appendix. CD3^+^ T cells within splenic T cell zones expressed lower levels of CD37 and CD53 compared to T cells in the red pulp of human spleen. B cells in human bone marrow highly expressed CD37, whereas the expression of CD53 was low. In conclusion, we demonstrate differential expression of CD37 and CD53 on primary human immune cells, their subcellular localization and their quantitative distribution in human lymphoid organs. This study provides a solid basis for better insight into the function of tetraspanins in the human immune response.

**Electronic supplementary material:**

The online version of this article (doi:10.1007/s00418-015-1326-2) contains supplementary material, which is available to authorized users.

## Introduction

The specific cellular architecture of primary and secondary lymphoid organs aids in the complex coordination of the initiation of the immune response against a wide variety of pathogens and tumor cells. The microarchitecture of lymphoid organs is highly dynamic, and its structure changes constantly upon antigen encountering. Whereas primary lymphoid organs (bone marrow, thymus) are the major sites of lymphocyte development, secondary lymphoid organs (spleen, lymph nodes and mucosal-associated lymphoid tissues, like appendix) provide a sophisticated environment in which immune cells interact with each other, as well as with accessory cells and antigens. Secondary lymphoid organs have a remarkably similar microanatomy under homeostatic conditions in which essentially three different regions can be distinguished (Junt et al. 2008). The outermost region is the antigen-sampling region where antigen-presenting cells, including macrophages and dendritic cells (DCs), sample and deliver antigens to the B and T cell areas. The outer cortex contains predominantly B cell follicles where germinal centers (GCs) can develop and the inner cortex comprises mainly T cells. A specialized conduit system, formed by interconnected fibroblastic reticular cells, supports migration of DCs and lymphocytes through different areas of the lymphoid organs to interact with other immune cells. In addition, this system provides a tubular network for distribution of small soluble antigens and immune modulators enabling communication between the antigen-sampling zone and the T cell zone (Roozendaal et al. 2008).

Cell–cell communication and immune cell migration are controlled by a wide variety of different immunoreceptors embedded in the plasma membrane. These immunoreceptors are non-randomly distributed in the plasma membrane by means of specialized membrane domains (Zuidscherwoude et al. 2014). This compartmentalization is essential for immune cell function, including pathogen recognition, antigen presentation and migration. Tetraspanin-enriched microdomains (TEM) are functional multimolecular complexes in the plasma membrane (Rubinstein et al. 1996; Hemler 2005; Yanez-Mo et al. 2009; Charrin et al. 2014) in which tetraspanins interact with each other and with partner molecules, like transmembrane immunoreceptors, enzymes and signaling proteins. Tetraspanin proteins belong to the superfamily of four-transmembrane proteins that are expressed at the cell surface and in intracellular membranes. To date, 33 different tetraspanins have been characterized in humans of which tetraspanins CD37 and CD53 are specific for the immune system (Hemler 2005). Tetraspanin CD37 has been studied extensively in CD37-deficient (*Cd37*−*/*−) mice in which both the cellular and humoral arms of the immune system are defective. CD37 inhibits T cell proliferation (van Spriel et al. 2004), interleukin-6 production by macrophages (Meyer-Wentrup et al. 2007) and antigen presentation by DCs (Sheng et al. 2009). Moreover, CD37 tightly regulates antibody production by B cells (van Spriel et al. 2012). *Cd37*−*/*− mice have a strikingly increased number of IgA+ plasma cells in their lymphoid organs, whereas the number of IgG+ plasma cells is low due to decreased survival signals in the GCs of the spleen (van Spriel et al. 2009, 2012). The function of tetraspanin CD53 has not been well studied although recurrent infections have been reported in a CD53-deficient family (Mollinedo et al. 1997). The underlying mechanism of tetraspanin function has been attributed to specific interactions between tetraspanins and immunoreceptors [major histocompatibility complex (MHC) proteins, B cell receptor (BCR), integrins and others] in the plasma membrane.

Despite the importance of tetraspanins in the immune system, little is known about their expression and microanatomical location. In this study, tetraspanins CD37 and CD53 were selected as these are among all tetraspanins exclusively present in the immune system. We report their membrane and intracellular expression on primary human blood immune cell subsets. Furthermore, we performed detailed quantitative immunohistochemical analyses using multispectral imaging to reveal tetraspanin expression and localization in human primary and secondary lymphoid organs. This novel technique allows for a direct unbiased overview of different tissues with the main advantage that, next to information on the single-cell expression level, the differential tissue localization of individual cell subsets can be studied.

## Materials and methods

### Cells

Cells were obtained from buffy coats of healthy individuals with informed consent in accordance with institutional and international guidelines following the Declaration of Helsinki. Peripheral blood mononuclear cells (PBMCs) were obtained by Ficoll density centrifugation. PBMCs were cultured in RPMI-1640 supplemented with 2 % human serum (HS) in Costar culture flasks (1 × 10^8^ cells/75 cm^2^ flask) to adhere monocytes for 1 h at 37 °C. After removal of peripheral blood lymphocytes (PBLs), monocytes were harvested with cold PBS.

### Flow cytometry

Single-cell suspensions were first stained with primary antibodies against human CD53 (mem53, Serotec), CD37 (WR17, home-made) or isotype controls in PBS with 1 % BSA and 0.05 % NaN_3_ (PBA) supplemented with 2 % HS for 30 min at 4 °C, followed by incubation with goat-anti-mouse Alexa488 antibody (Molecular Probes). Next, cells were stained with the following anti-human antibodies: CD3-PE (HIT3a, Becton–Dickinson), CD4-APC (RPA-T4, Biolegend), CD8-PerCP (SK1, Becton–Dickinson), CD20-APC (2H7, eBioscience), CD14-PE (CLB-mon/1, Pelicluster, Sanquin), CD56-APC (NCAM16.2, Becton–Dickinson), CD19-PerCP (4G7, Becton–Dickinson), BDCA1-FITC (AD5-8E7, Miltenyi) and/or BDCA2-FITC (AC144, Miltenyi). Stained cells were analyzed using FACS Calibur (Becton–Dickinson) and FlowJo software (version 9.7, TreeStar Inc.).

### Confocal microscopy

Fibronectin-coated coverslips were made by incubation of 20 μg/mL fibronectin (Roche) in PBS for 1 h at 37 °C. Monocytes were adhered on fibronectin-coated coverslips for 2 h and subsequently fixed with 2 % paraformaldehyde (PFA) and blocked with 3 % bovine serum albumin (BSA), 1 % HS and 10 mM glycine in PBS for 30 min at room temperature (RT). Cells were permeabilized and stained with antibodies against CD53 (mem53, Serotec), CD37 (WR17, home-made), calreticulin (ER marker, Sigma), syntaxin 12/13 [endosome marker, Synaptic Systems (cat. no. 110132)] and Lamp1 (lysosome marker, Sigma-Aldrich) in 0.5 % saponin, 1 % BSA, 10 mM glycine, 1 % HS in PBS, followed by goat-anti-mouse Alexa488 and goat-anti-rabbit Alexa647 (Molecular Probes). Samples were imaged with an Olympus FV1000 confocal laser scanning microscope. Images were analyzed using Fiji software (Schindelin et al. 2012).

### Tissues

Human spleen samples were obtained from deceased human kidney donors and bone marrow, and appendix samples were obtained from healthy donors, which were approved by the Medical Ethical Committee for Human Research (Radboudumc, The Netherlands). All tissue samples were formalin-fixed, paraffin-embedded and cut in 4 μm sections according to standard procedures (Canene-Adams 2013).

### Immunofluorescence

Tissues were deparaffinized, followed by antigen retrieval using 10 mM citrate buffer and blocked with 10 % normal goat serum (NGS) in PBA for 1 h at RT. Tissues were first stained with antibodies against human CD37 (clone 2B8, Thermo Scientific) or CD53 (clone EPR4342(2), GenTex) diluted in 2 % NGS in PBA for 1 h at RT, followed by incubation with Alexa-conjugated secondary antibodies diluted in 1 % NGS in PBA for 1 h at RT. For nuclear staining, tissues were incubated for 5 min with DAPI (diluted 1:3000 in PBS). Tissue slides were fixed in 1 % PFA in PBS for 15 min at RT and embedded in Mowiol mounting medium.

### Immunohistochemistry

Tissues were deparaffinized, followed by antigen retrieval using 10 mM citrate buffer and blocked with 3 % hydrogen peroxidase (H_2_O_2_) in methanol for 10 min at RT. Tissues were blocked with 2 % HS in Tris-buffered saline (TBS) with 1 % BSA for 45 min at RT and stained with primary antibodies against human CD3 (clone CD3-12, AbD Serotec) or CD20 (clone L26, Thermo Scientific) diluted in TBS supplemented with 1 % BSA overnight at 4 °C. Next, tissues were incubated with biotinylated secondary antibodies diluted in TBS with 1 % BSA, 1 % HS and 1 % NGS or normal horse serum (NHS) for 45 min at RT. After 1 h incubation with avidin–biotin alkaline phosphatase complex (ABC-AP) solution, red color was developed by incubation with Warp Red Solution for 10 min. Secondly, tissues were stained with antibodies against human CD37 (clone 2B8, Thermo Scientific) or CD53 (clone EPR4342(2), GenTex) diluted in PBS, 1 % BSA, 2 % HS for 45 min at RT, followed by incubation with biotinylated secondary antibodies in TBS with 1 % BSA, 1 % HS and 1 % NHS or NGS for 45 min at RT. After 1 h incubation with avidin–biotin horseradish peroxidase complex (ABC-HRP) solution, blue color was developed by incubation with True Blue peroxidase substrate for 8 min. For technical reasons, CD20 and CD37 could not be stained on the same slide, as such single staining for CD37 was performed. For nuclear staining, tissues were incubated for 1 min with Nuclear Red. Dried sections were embedded in Permount.

### Multispectral imaging and quantitative digital analysis

Tissue slides were imaged using Vectra Intelligent Slide Analysis System (version 2.0.8, PerkinElmer Inc.). This imaging technique combines imaging with spectroscopy where the entire spectrum is collected at every location of the image plane in an automatic manner. Images of single-stained tissues for each fluorophore or chromogen, with its own unique spectral characteristics, were used to built spectral libraries with Nuance Multispectral Imaging System (version 3.0.2, PerkinElmer Inc.). For correction of autofluorescence, an image was made from unstained human spleen tissue and the autofluorescence signal was subtracted from the spectrum for each fluorophore. These spectral libraries were used to unmix the original multispectral images obtained with the Vectra imaging system. Two red chromogens (Warp Red and Nuclear Red) with highly similar spectra were used of which correct unmixing has been described before (Van Der Loos 2010). A selection of ten representative original multispectral images was used to train the inForm Advanced Image Analysis Software (version 2.0.2, PerkinElmer Inc.) for quantitative image analysis (tissue and cell segmentation, quantification of the expression levels of proteins of interest) as described before (Mansfield 2010; Stack et al. 2014). The threshold for dim or bright tetraspanin expression was determined by objective examination of the staining intensity of non-immune cells in True Blue-negative regions. All the settings applied to the training images were saved within an algorithm allowing batch analysis of multiple original multispectral images of different samples of the same origin equally stained. The number of images used for quantification and statistical analysis of CD37 and CD53 distribution was dependent on the acquisition of the tissue slides during imaging (i.e., the position of the tissue on the coverslip can vary, leading to acquisition of less or more images). If 20× images contained less than 10 % of tissue, these images were discarded from the analysis. Since not all 20× images contained B cell follicles or T cell regions, a higher number of images were analyzed for red pulp or lamina propria regions (Supplementary Table 1).

### Statistics

Statistical differences of tetraspanin expression between different tissue regions in human lymphoid organs were determined using the unpaired Student’s *t* test or, in case of a non-Gaussian distribution, the Mann–Whitney test (GraphPad Prism 5, GraphPad Software, San Diego, CA, USA). All differences with *P* ≤ 0.05 were considered to be statistically significant.

## Results

### Expression of CD37 and CD53 on immune cell subsets in blood

**Fig. 1:**
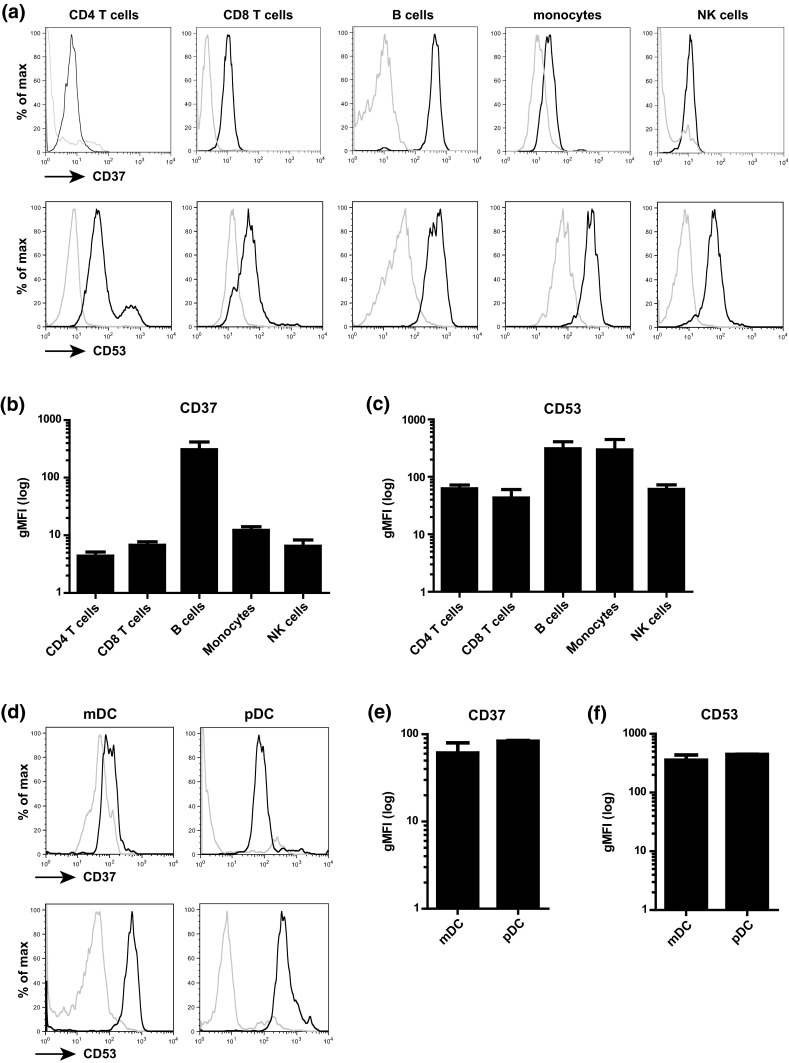
Expression of CD37 and CD53 on immune cell subsets in blood. **a** Flow cytometry analysis of expression of CD37 or CD53 (*black line*) on CD4 and CD8 T cells, B cells, monocytes and NK cells versus isotype control (*gray line*). Gating strategy is presented in Supplementary Figure 1. Expression levels of CD37 (**b**) and CD53 (**c**) were normalized for isotype staining by background subtraction. Experiments were performed with PBLs from three healthy donors. Data present mean ± SD. **d** Flow cytometry analysis of expression of CD37 or CD53 (*black line*) on mDCs (BDCA1^+^CD19^−^) or pDCs (BDCA2^+^) versus isotype control (*gray line*). Expression levels of CD37 (**e**) and CD53 (**f**) were normalized for isotype staining by background subtraction. Experiments were performed with PBLs from two healthy donors. Data present mean ± SD

**Fig. 2:**
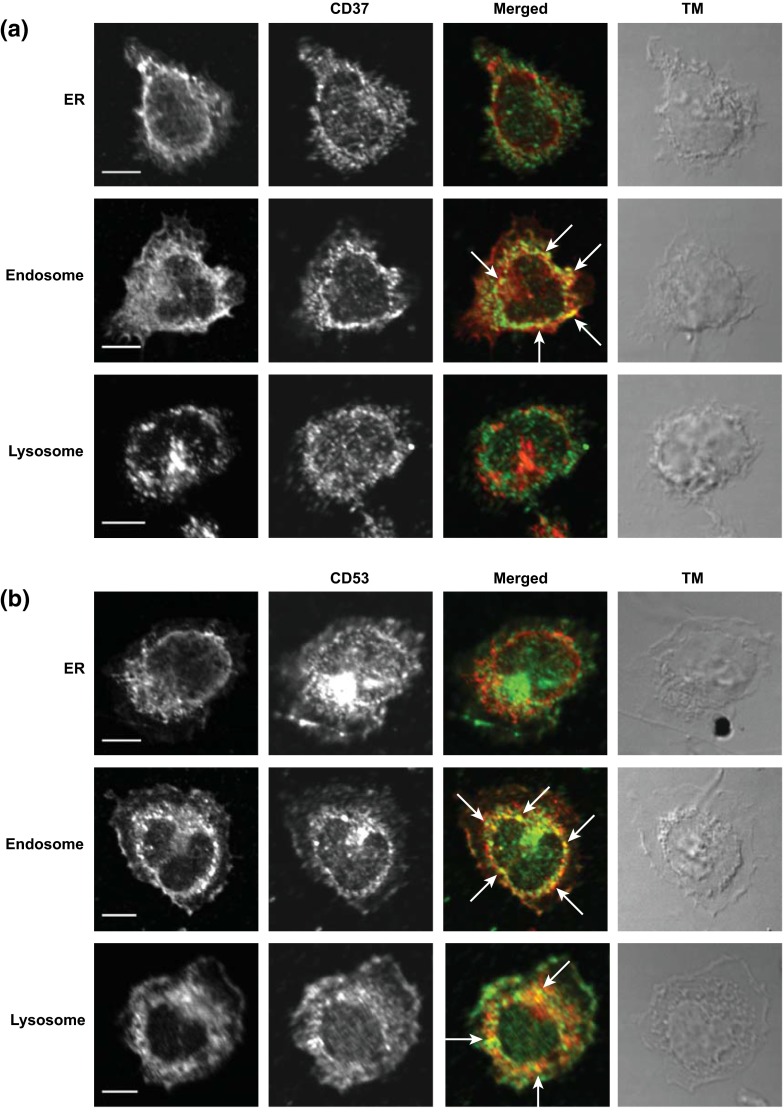
Subcellular localization of CD37 and CD53. Localization of **a** CD37 or **b** CD53 (*green*) in monocytes was studied by dual staining with calreticulin (ER), syntaxin 13 (endosomes) or Lamp1 (lysosomes) (*red*). Merge: co-localization in *yellow* (*white arrows*). *Scale bar* 5 μm

To investigate cell surface expression of tetraspanins CD37 and CD53 on the plasma membrane of different immune cell subsets in blood, PBLs were stained for CD4 (T cells), CD8/CD3/CD56− (T cells), CD20 (B cells), CD14 (monocytes), CD56/CD3−/CD8− (NK cells) and BDCA1/CD19− (myeloid DC (mDC)) and BDCA2 (plasmacytoid DC (pDC)) (Supplementary Fig. 1). We observed the highest CD37 expression on B cells and low to medium expression on T cells, monocytes and NK cells (Fig. 1a, b). CD53 was expressed on all subsets, with highest expression on B cells and monocytes (Fig. 1a, c). CD37 and CD53 were expressed on both mDCs and pDCs, with no apparent differences in expression level between the two DC subsets (Fig. 1d–f). It has been reported that tetraspanins can be expressed at intracellular membranes (Kobayashi et al. 2000; Xu et al. 2009), which stimulated us to investigate the subcellular localization of CD37 and CD53. Monocytes were double stained with CD37 or CD53 antibodies in combination with antibodies specific for the endoplasmatic reticulum (ER), endosomes or lysosomes. Next to the expression on the plasma membrane, both CD37 and CD53 were abundantly expressed in the endosomes, in contrast to the ER (Fig. 2a, b). In the lysosomes, we observed only CD53 to be present.

### Multispectral analyses of human lymphoid organs

**Fig. 3:**
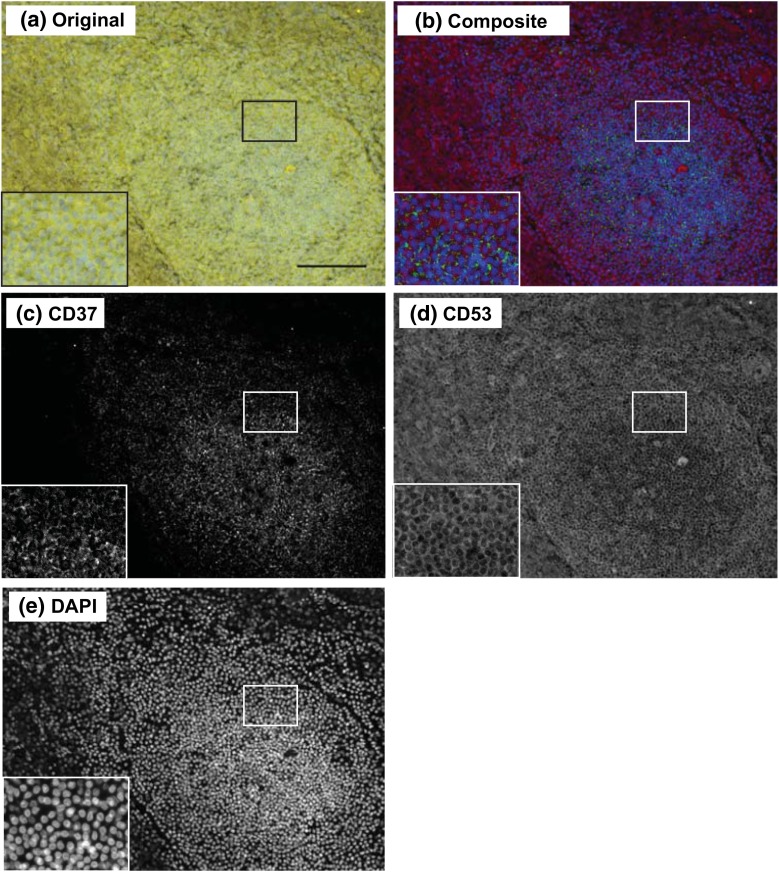
Expression of CD37 and CD53 on human spleen. **a** Original multispectral image of human spleen stained for CD37 (Alexa488), CD53 (Alexa568) and cell nuclei (DAPI). *Scale bar* 100 μm. Composite RGB image (**b**) of unmixed CD37 (**c** in *green*), CD53 (**d** in *red*) and DAPI (**e** in *blue*) signal after correction for autofluorescence. One representative image is shown

**Fig. 4:**
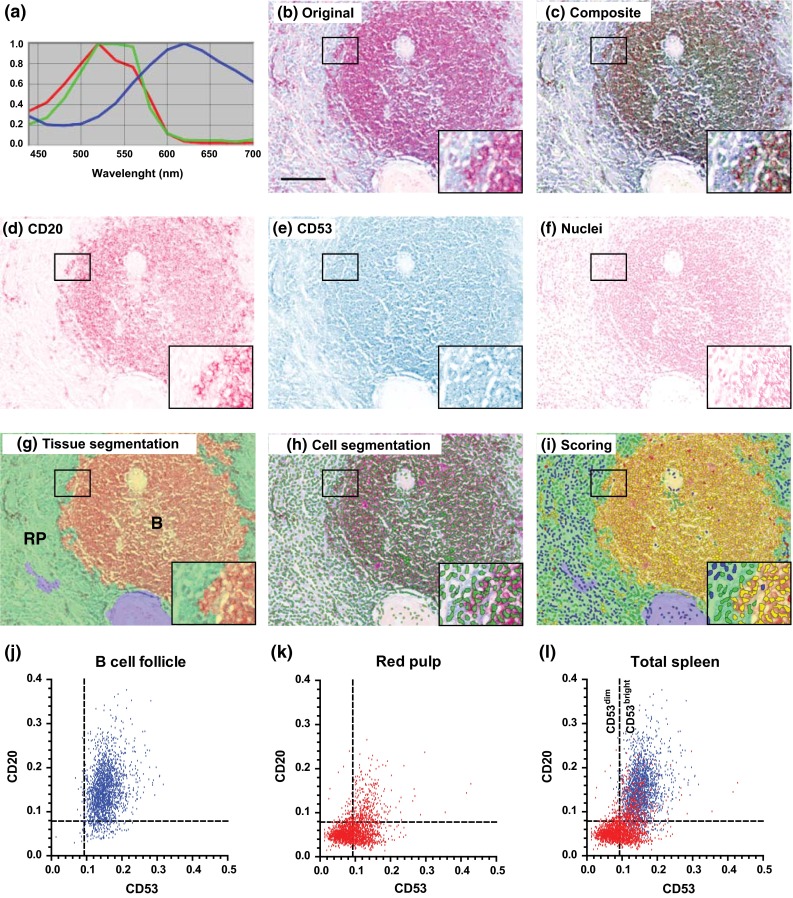
Spectral imaging analysis of human spleen stained for CD20 (Warp Red), CD53 (True Blue) and cell nuclei (Nuclear Red). **a** A spectral library of three chromogens (Warp Red (*red line*), True Blue (*blue line*) and Nuclear Red (*green line*)) was built in Nuance software using single-stained human spleen tissues. **b** Representative original multispectral image. *Scale bar* 100 μm. Composite RGB image (**c**) of unmixed CD20 (**d** in *red*), CD53 (**e** in *blue*) and nuclei (**f** in *green* in composite RGB image) signal. **g** Tissue segmentation; B cell follicle (B, *yellow*), red pulp (RP, *green*) and other tissue (blood vessels, collagen; *blue*). **h** Segmentation of individual cells (*green*) based on Nuclear Red staining. **i** Thresholds for Warp Red and True Blue staining were set to score CD20^−^CD53^dim^ (*blue*), CD20^+^CD53^dim^ (*red*), CD20^+^CD53^bright^ (*yellow*) or CD20^−^CD53^bright^ (*green*) cells. **j–l** Scatter plots showing optical densities for CD20 (Y-axis) and CD53 (X-axis) of individual cells in B cell follicles (**j**, **l** *blue*) and red pulp (**k–l** *red*) and thresholds used for scoring (*dotted lines*). A representative of 2000 cells per tissue region is plotted

**Fig. 5:**
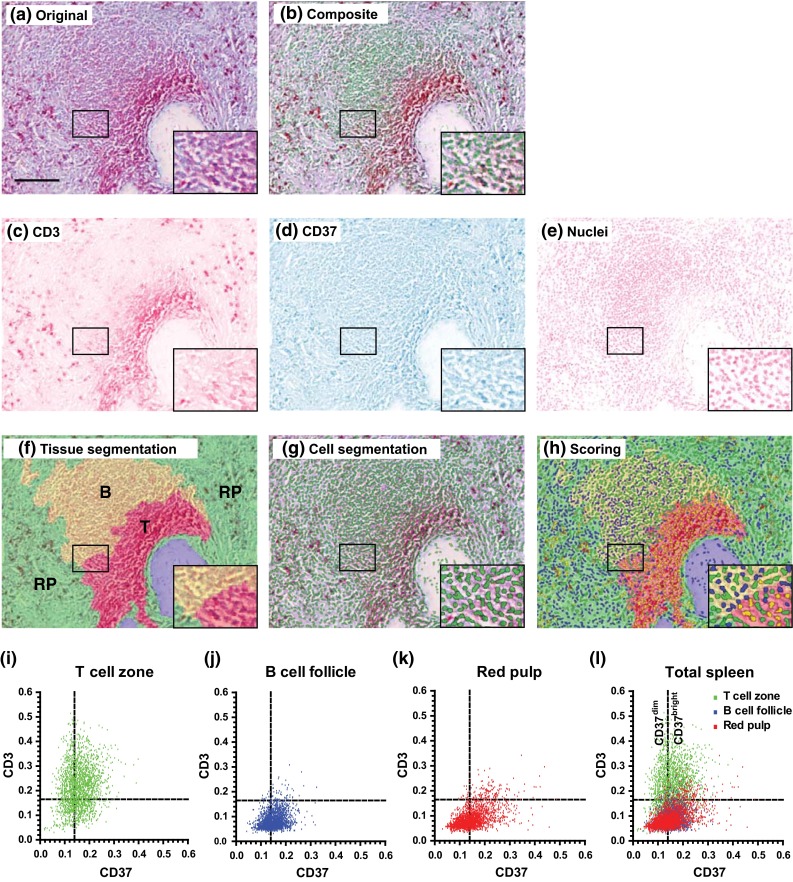
Spectral imaging analysis of human spleen stained for CD3 (Warp Red), CD37 (True Blue) and cell nuclei (Nuclear Red). **a** Representative original multispectral image. *Scale bar* = 100 μm. Composite RGB image (**b**) of unmixed CD3 (**c** in *red*), CD37 (**d** in *blue*) and Nuclear Red (**e** in *green* in composite RGB image) signal using the spectral library (Fig. 4a). **f** Tissue segmentation; B cell follicle (B, *yellow*), T cell zone (T, *red*), red pulp (RP, *green*) and other tissue (blood vessels, collagen; *blue*). **g** Segmentation of individual cells (*green*) based on Nuclear Red staining. **h** Thresholds for Warp Red and True Blue staining were set to score CD3^−^CD37^dim^ (*blue*), CD3^+^CD37^dim^ (*red*), CD3^+^CD37^bright^ (*yellow*) or CD3^−^CD37^bright^ (*green*) cells. **i–l** Scatter plots showing optical densities for CD3 (Y-axis) and CD37 (X-axis) of individual cells in T cell zones (**i**, **l** *green*), B cell follicles (**j**, **l** *blue*) and red pulp regions (**k–l** *red*) and thresholds used for scoring (*dotted lines*). A representative of 2000 cells per tissue region is plotted

We investigated the tissue distribution of CD37 and CD53 in human lymphoid organs by multispectral imaging. In contrast to classical immunohistochemistry, multispectral imaging directly provides quantitative information into the differential tissue distribution of individual cell subsets. First, we investigated localization of CD37 and CD53 in human spleen. We observed that CD37 was more locally expressed in follicle-like structures when compared to CD53 which showed a more dispersed expression profile (Fig. 3a–e). To explore this in more detail, we performed double staining of either the T cell marker CD3 or the B cell marker CD20 combined with CD37 or CD53 on primary (bone marrow) and secondary (spleen and appendix) lymphoid tissues. Figure 4 illustrates the technology of multispectral imaging and analysis of lymphoid tissue stained for the B cell marker CD20 (Warp Red), tetraspanin CD53 (True Blue) and cell nuclei (Nuclear Red). Single-stained tissues for each chromogen (Warp Red, True Blue and Nuclear Red) were used to create a spectral library containing the specific spectra of each used chromogen (Fig. 4a) allowing to unmix the original multispectral images (Fig. 4b). This resulted in separate images for each marker (Fig. 4d–f) that were used to generate the composite RGB image (Fig. 4c). We made use of two red chromogens (Warp Red and Nuclear Red) with highly similar spectra of which correct unmixing has been described before (Van Der Loos 2010). Next, analysis software was trained using ten representative original multispectral images to segment the different tissue regions (B cell follicle and stromal tissue (red pulp in spleen or lamina propria in appendix)) based on a combination of parameters including cell morphology and specific staining (Fig. 4g) and individual cells based on nuclear characteristics (Fig. 4h). For each cell, CD20 positivity and CD53 expression were determined in relation to tissue localization (Fig. 4i–l). These settings were saved within an algorithm allowing batch analysis of multiple original multispectral images of the same tissue and stainings. Figure 5 shows similar analysis for lymphoid tissue stained for the T cell marker CD3 (Warp Red), tetraspanin CD37 (True Blue) and cell nuclei (Nuclear Red). Original multispectral images were unmixed using the spectral library showed in Fig. 4a (Fig. 5a–e). Next, tissue segmentation was performed for T cell zones, B cell follicles and red pulp regions (Fig. 5f), followed by cell segmentation (Fig. 5g) and analysis of CD3 and CD37 expression within the different tissue regions (Fig. 5h–l). As expected, B cell follicles mainly consisted of CD20-positive cells, and T cell zones contained mainly CD3-positive cells. The stromal tissue consisted of both CD20- or CD3-negative and CD20- or CD3-positive cells. Altogether, we established multispectral imaging analysis to combine quantitative tetraspanin expression data with specific tissue localization in human lymphoid tissues.

### Localization and quantitative expression of CD37 and CD53 in lymphoid organs

**Fig. 6:**
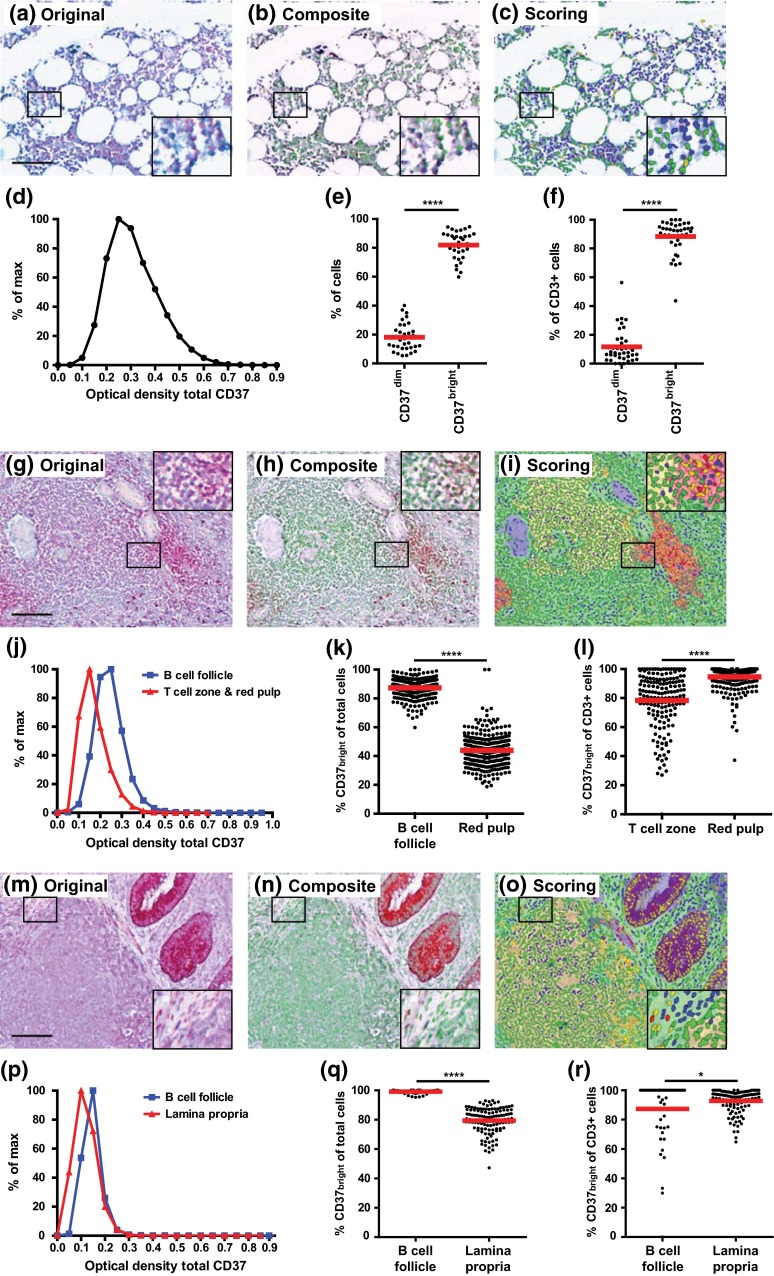
Localization and expression of CD37 in bone marrow (**a–f**), spleen (**g–l**) and appendix (**m–r**). **a**, **g**, **m** Representative original multispectral image of lymphoid organ stained for CD3 (Warp Red), CD37 (True Blue) and cell nuclei (Nuclear Red). *Scale bars* = 100 μm. **b**, **h**, **n** Composite RGB image after spectral unmixing of original image (*red* CD3, *blue* CD37, *green* nuclei). **c**, **i**, **o** Image showing scoring of CD3^−^CD37^dim^ (*blue*), CD3^+^CD37^dim^ (*red*), CD3^+^CD37^bright^ (*yellow*) or CD3^−^CD37^bright^ (*green*) cells. Optical density of CD37 on individual cells in human bone marrow (**d**), in B cell follicle (*blue line*) and in T cell zone and in red pulp (*red line*) in human spleen (**j**) and in B cell follicle (*blue line*) and in lamina propria (*red line*) in human appendix (**p**). Optical densities were binned per 0.05 and normalized to % of max. Percentage of CD37^dim^ and CD37^bright^ cells in total bone marrow (**e**) and in the CD3^+^ cell population in human bone marrow (**f**). Percentage of total CD37^bright^ cells in B cell follicle and red pulp (**k**) and in the CD3^+^ cell population in T cell zone and red pulp (**l**) in human spleen. Percentage of total CD37^bright^ cells (**q**) and in the CD3^+^ cell population (**r**) in B cell follicle and lamina propria in human appendix. Each *dot* represents data of one ×20 image from the lymphoid tissue. The *red line* represents the mean. *P < 0.05, ****P < 0.0001

**Fig. 7:**
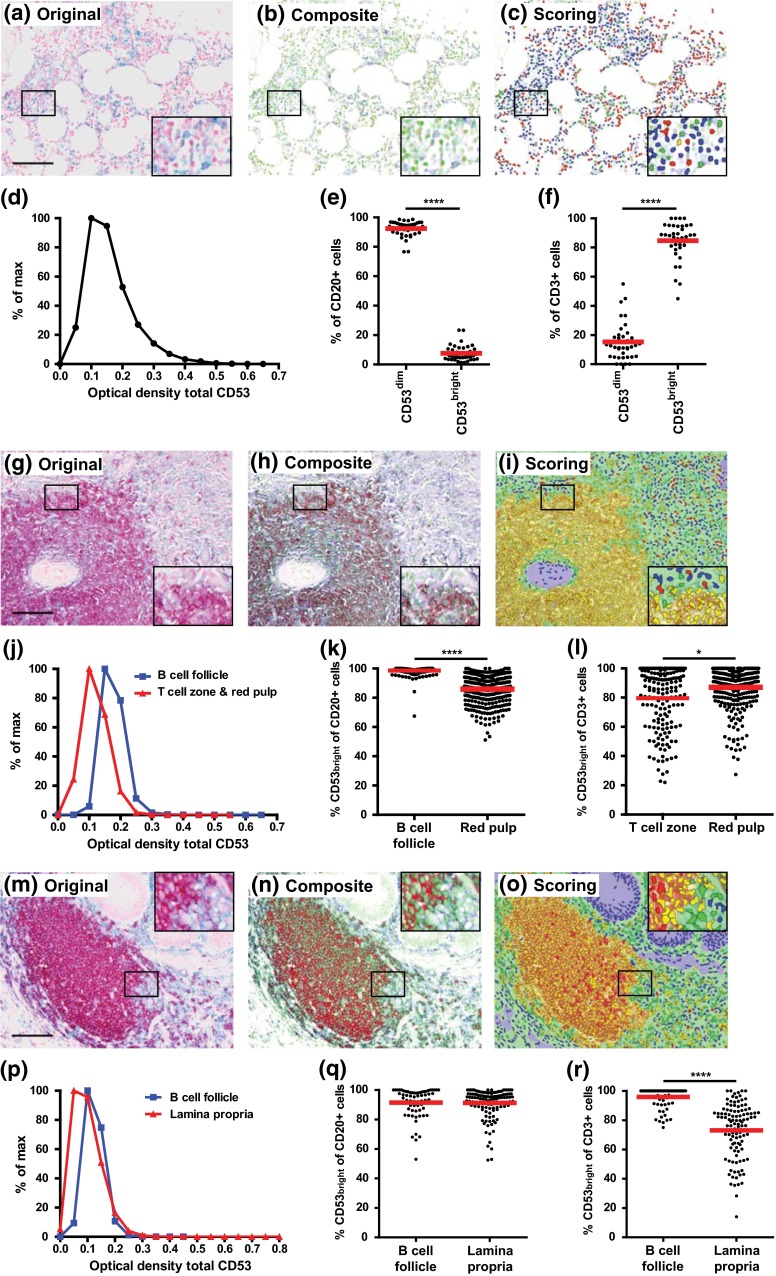
Localization and expression of CD53 in bone marrow (**a–f**), spleen (**g–l**) and appendix (**m–r**). **a**, **g**, **m** Representative original multispectral image of lymphoid organ stained for CD20 (Warp Red), CD53 (True Blue) and cell nuclei (Nuclear Red). *Scale bars* in **a**, **g**, **m** = 100 μm. **b**, **h**, **n**, Composite RGB image after spectral unmixing of original image (*red* CD20, *blue* CD53, *green* nuclei). **c**, **i**, **o**, Image showing scoring of CD20^−^CD53^dim^ (*blue*), CD20^+^CD53^dim^ (*red*), CD20^+^CD53^bright^ (*yellow*) or CD20^−^CD53^bright^ (*green*) cells. **d**, **j**, **p**, Optical density of CD53 on individual cells in human bone marrow (**d**), in B cell follicle (*blue line*) and in T cell zone and in red pulp (*red line*) in human spleen (**j**) and in B cell follicle (*blue line*) and in lamina propria (*red line*) in human appendix (**p**). Optical densities were binned per 0.05 and normalized to % of max. Percentage of CD53^dim^ and CD53^bright^ cells in the CD20^+^ cell population (**e**) and in the CD3^+^ cell population (**f**) in human bone marrow. Percentage of CD53^bright^ cells in the CD20^+^ cell population in the B cell follicle and red pulp (**k**), and in the CD3^+^ cell population in the T cell zone and red pulp (**l**) in human spleen. Percentage of CD53^bright^ cells in the CD20+ cell population (**q**) and in the CD3^+^ cell population (**r**) in the B cell follicle and lamina propria in human appendix. Each *dot* represents data of one ×20 image from the lymphoid tissue. The *red line* represents the mean. *P < 0.05, ****P < 0.0001

We studied the localization and expression of tetraspanin CD37 in primary and secondary lymphoid organs. Since bone marrow does not contain different T and B cell areas, tissue segmentation was not applicable and only cell segmentation was performed (Figs. 6a–c, 7a–c). The intensity [optical density (OD)] of CD37 in bone marrow ranged from 0.05 to 0.85, with a mean of 0.29 (OD_mean_; Fig. 6d). We observed around 80 % of all bone marrow cells to be CD37^bright^ (Fig. 6e) and 90 % of all T cells to be highly positive for CD37 (Fig. 6f). The scatter plots with the set thresholds to annotate cells with dim and bright expression of CD37 and CD53 are shown in Figs. 4j–l, 5i–l and Supplementary Figures 2 and 3. In human spleen, B cell follicles, T cell zones and red pulp areas were efficiently distinguished (Figs. 6g–i, 7g–i). CD37 showed highest expression in the B cell follicles (OD_mean_ = 0.22) compared to the T cell zones and red pulp areas (OD_mean_ = 0.15; Fig. 6j). In splenic B cell follicles, twice as many cells were CD37^bright^, compared to the red pulp where only 45 % of the cells was CD37^bright^ (Fig. 6k). When focusing on CD37 expression on splenic T cells, we observed that significantly more T cells were CD37^bright^ in the red pulp as compared to T cells in T cell zones (Fig. 6l). In the appendix, B cell follicles and lamina propria regions were located immediately below the crypts (Figs. 6m–o, 7m–o). CD37 showed highest expression in B cell follicles (OD_mean_ = 0.12) compared to the lamina propria (OD_mean_ = 0.09) in appendix (Fig. 6p) which is in line with CD37 expression in spleen. In the B cell follicles in human appendix, almost all cells were CD37^bright^, which was significantly more than in the lamina propria where around 80 % of total cells expressed high levels of CD37 (Fig. 6q). Similar to the red pulp in spleen, significantly more T cells in the lamina propria were CD37^bright^ compared to the T cells within B cell follicles of the appendix (Fig. 6r). However, we need to be careful with interpreting these data, because the frequency of T cells in the appendix is very low.

Next, we performed multispectral imaging analyses for tetraspanin CD53 in human bone marrow, spleen and appendix. In bone marrow, the OD of CD53 ranged between 0.05 and 0.60, with an OD_mean_ of 0.13 (Fig. 7d). The CD20^+^ B cells in bone marrow were mostly CD53^dim^ (Fig. 7e), contrary to the majority of CD3^+^ T cells that were CD53^bright^ (Fig. 7f). In human spleen, we observed CD53 to be expressed at higher levels in B cell follicles (OD_mean_ = 0.15) than in the red pulp and T cell zones (OD_mean_ = 0.09; Fig. 7j). In contrast to human bone marrow, almost 100 % of CD20^+^ B cells in the spleen were CD53^bright^, significantly more than in the splenic red pulp where 85 % of B cells expressed high levels of CD53 (Fig. 7k). Similar to CD37 expression, significantly more T cells in the red pulp compared to the T cell zone were CD53^bright^ (Fig. 7l). In human appendix, CD53 was slightly higher expressed in B cell follicles (OD_mean_ = 0.10) than in the lamina propria regions (OD_mean_ = 0.07; Fig. 7p). B cells within B cell follicles and lamina propria expressed similar levels of CD53 (Fig. 7q), whereas significantly more T cells in the B cell follicle expressed high levels of CD53 compared to T cells in the lamina propria (Fig. 7r). Together, these data demonstrate that CD37 and CD53 are differentially localized and expressed in human bone marrow and in B cell, T cell and red pulp or lamina propria regions in human spleen and appendix. Furthermore, B and T cells have different expression levels of tetraspanin proteins depending on their localization within the tissue.

## Discussion

Here, we provide the quantitative expression of tetraspanins directly linked to tissue distribution in human lymphoid organs using multispectral imaging. Tetraspanin proteins are important in controlling cellular immune responses which is evidenced by the defects in the immune system of different tetraspanin-deficient mice (Knobeloch et al. 2000; Takeda et al. 2008; Sheng et al. 2009; Gartlan et al. 2010; Kraft et al. 2013).

We demonstrate abundant expression of CD53 on lysosomes and of both tetraspanins on endosomes in human monocytes. The presence of tetraspanin proteins in intracellular vesicles has been linked to their function to regulate the trafficking of their partner proteins through the cell (Berditchevski and Odintsova 2007; Charrin et al. 2014). In B cells, both CD37 and CD53 can be found in multivesicular endosomes, called MHC class II-enriched compartments (Escola et al. 1998). Furthermore, it has been described that CD37 contains a tyrosine-based sorting motif which targets this protein to endocytic vesicles (Berditchevski and Odintsova 2007).

The predominant expression of CD37 observed on B lymphocytes and in B cell follicles is in line with former studies reporting an important role for CD37 in B cell function (Knobeloch et al. 2000; van Spriel et al. 2009, 2012; Lapalombella et al. 2012). In the cytoplasmic tails of CD37, “ITIM-like” and “ITAM-like” motifs have been reported that regulate B cell death and survival, respectively (Lapalombella et al. 2012). Interestingly, the dynamic process of B cell differentiation and selection in GCs coincides with lower CD37 expression toward plasma cell differentiation (Barrena et al. 2005). Tetraspanins play a well-established role in cancer development and progression (Hemler 2014), and CD37-directed targeted therapies are currently under investigation in clinical trials in patients with B cell malignancies (Zhao et al. 2007; Robak et al. 2009; Rafiq et al. 2013).

We observed primary myeloid and plasmacytoid DCs in human blood to express CD37 protein suggesting that CD37 may be involved in human DC function. This is supported by studies with DCs of CD37-deficient mice which show that CD37 promotes cell migration (Gartlan et al. 2013) and inhibits antigen presentation via MHC class II molecules (Sheng et al. 2009). T cells present in lymphoid organs were mostly CD37^bright^, which may be linked to the reported function of CD37 in T cell proliferation, which predominantly takes place in lymphoid organs upon antigen presentation by DCs. T cells that are deficient in CD37 have disturbed regulation of T cell receptor signaling leading to increased proliferation (van Spriel et al. 2004). Remarkably, lymphocytes in various tissue regions within spleen and appendix often expressed significantly different levels of CD37 and CD53. For example, significantly more T cells in the red pulp were CD37^bright^ compared to T cells in the T cell zone, suggesting that CD37 expression correlates with immune cell localization though it is also possible that these represent different T cell subsets.

The role of CD53 in the immune system has not been clearly defined although a CD53-deficient family has been reported that suffered from recurrent infections (Mollinedo et al. 1997). Our study now demonstrates that CD53 in blood is expressed on all immune cells with the highest expression on B cells, monocytes and mDC and pDC subsets. Within the secondary lymphoid tissues spleen and appendix, CD53 was highly expressed on both CD20^+^ and CD3^+^ cells. Surprisingly, we found CD20^+^ B lymphocytes in bone marrow to be mostly CD53^dim^ which may be related to the finding that CD53 is under the control of the transcription factor early B cell factor-1 (EBF-1) (Månsson et al. 2007). EBF-1 is essential for B cell development by inducing expression of the genes encoding the (pre-)BCR, from which production is started in pre-B cells. CD53 has also been shown to interact with protein kinase C (PKC) (Zhang et al. 2001; personal communication), a central signaling molecule important in cell proliferation, differentiation and apoptosis. We anticipate that CD53 may only be expressed during later stages of B cell development in the bone marrow when the (pre-)BCR is expressed, which is in line with an earlier study showing lower levels of CD53 in earlier maturation stages of B cells within bone marrow (Barrena et al. 2005). Furthermore, we demonstrate abundant CD53 expression on T cells within blood and lymphoid organs. Although CD53 function in T cells is largely unknown to date, a strong correlation between CD53 expression on murine thymocytes and positive selection has been reported in the thymus (Puls et al. 2002).

In conclusion, we demonstrate the differential expression of tetraspanins CD37 and CD53 in the human immune system. Multispectral imaging allowed us to obtain quantitative expression data that are directly linked to tissue distribution. This study offers guidance for further exploring tetraspanin function in the human immune system using this novel imaging technique.

## Electronic supplementary material

Supplementary material 1 (DOCX 14 kb)

Supplementary material 2 (DOCX 13 kb)

Supplementary material 3 (PDF 2123 kb)
