# Supplementary material for: Multispectral imaging reveals the tissue distribution of tetraspanins in human lymphoid organs
Source: Histochem Cell Biol. 2015 May 8;144(2):133–46. doi: 10.1007/s00418-015-1326-2 (PMC4522275; doi:10.1007/s00418-015-1326-2)
Supplement: Supplementary file 1 — Supplementary material 1 (DOCX 14 kb) [file 418_2015_1326_MOESM1_ESM.docx]

**Supplementary table**

**Table 1** Number of 20x images used for quantification and statistical analysis of CD37 and CD53 distribution in Figure 6 and 7.

| **Figure #** | **Organ** | **# of images used for quantification**  **and statistical analysis** | |
| --- | --- | --- | --- |
| 6e | Bone marrow | 35 | |
| 6f | Bone marrow | 40 | |
| 7e | Bone marrow | 41 | |
| 7f | Bone marrow | 38 | |
|  | | | |
|  |  | B cell follicle or T cell zone | Red pulp |
| 6k | Spleen | 296 | 311 |
| 6l | Spleen | 174 | 273 |
| 7k | Spleen | 233 | 325 |
| 7l | Spleen | 182 | 298 |
|  | | | |
|  |  | B cell follicle | Lamina propria |
| 6q | Appendix | 94 | 120 |
| 6r | Appendix | 50 | 112 |
| 7q | Appendix | 65 | 124 |
| 7r | Appendix | 69 | 107 |
