# Supplementary material for: Multispectral imaging reveals the tissue distribution of tetraspanins in human lymphoid organs
Source: Histochem Cell Biol. 2015 May 8;144(2):133–46. doi: 10.1007/s00418-015-1326-2 (PMC4522275; doi:10.1007/s00418-015-1326-2)
Supplement: Supplementary file 2 — Supplementary material 2 (DOCX 13 kb) [file 418_2015_1326_MOESM2_ESM.docx]

**Supplementary Information**

**Supplementary figure legends**

**Supplementary Fig. 1** Flow cytometry gating strategy of immune cell subsets in PBLs. **a-h**, live cells were gated on FSC-SSC plots (**a**). From the live cells, CD4^+^ T cells (**b**), CD8^+^ (CD3^+^CD56^-^) T cells (**c**), CD20^+^ B cells (**d**), CD14^+^ monocytes (**e**), CD56^+^ (CD3^-^CD8^-^) NK cells (**f**), BDCA1^+^CD19^-^ mDCs (**g**) and BDCA2^+^ pDCs (**h**) were gated.

**Supplementary Fig. 2** Scatter plots of CD3 and CD37 expression in human bone marrow, spleen and appendix. **a-b**, expression of CD3 and CD37 (**a**) and CD37 alone (**b**) in human bone marrow. **c**, expression of CD37 alone in human spleen (scatter plots of CD3 and CD37 shown in Fig. 5i-l). Blue = B cell follicle, red = red pulp & T cell zone. **d-e**, expression of CD3 and CD37 (**d**) and CD37 alone (**e**) in human appendix. Blue = B cell follicle, red = lamina propria. Dotted lines represent threshold used for analysis of scoring CD3 positive and negative cells (horizontal line) or CD37^dim^ and CD37^bright^ cells (vertical line) in inForm software. Data of 2000 representative cells per tissue region are shown.

**Supplementary Fig. 3** Scatter plots of CD3 or CD20 and CD53 expression in human bone marrow, spleen and appendix. **a-b**, expression of CD3 (**a**) or CD20 (**b**) and CD53 in human bone marrow. **c**, expression of CD3 and CD53 in human spleen (scatter plots of CD20 and CD53 shown in Fig. 4j-l). Green = T cell zone, blue = B cell follicle, red = red pulp. **d-e**, expression of CD3 (**d**) or CD20 (**e**) and CD53 in human appendix. Blue = B cell follicle, red = lamina propria. Dotted lines represent threshold used for analysis of scoring CD3 or CD20 positive and negative cells (horizontal line) or CD53^dim^ and CD53^bright^ cells (vertical line) in inForm software. Data of 2000 representative cells per tissue region are shown.
