# Supplementary figures and images for: Multispectral imaging reveals the tissue distribution of tetraspanins in human lymphoid organs
Source: Histochem Cell Biol. 2015 May 8;144(2):133–46. doi: 10.1007/s00418-015-1326-2 (PMC4522275; doi:10.1007/s00418-015-1326-2)

Figure S1

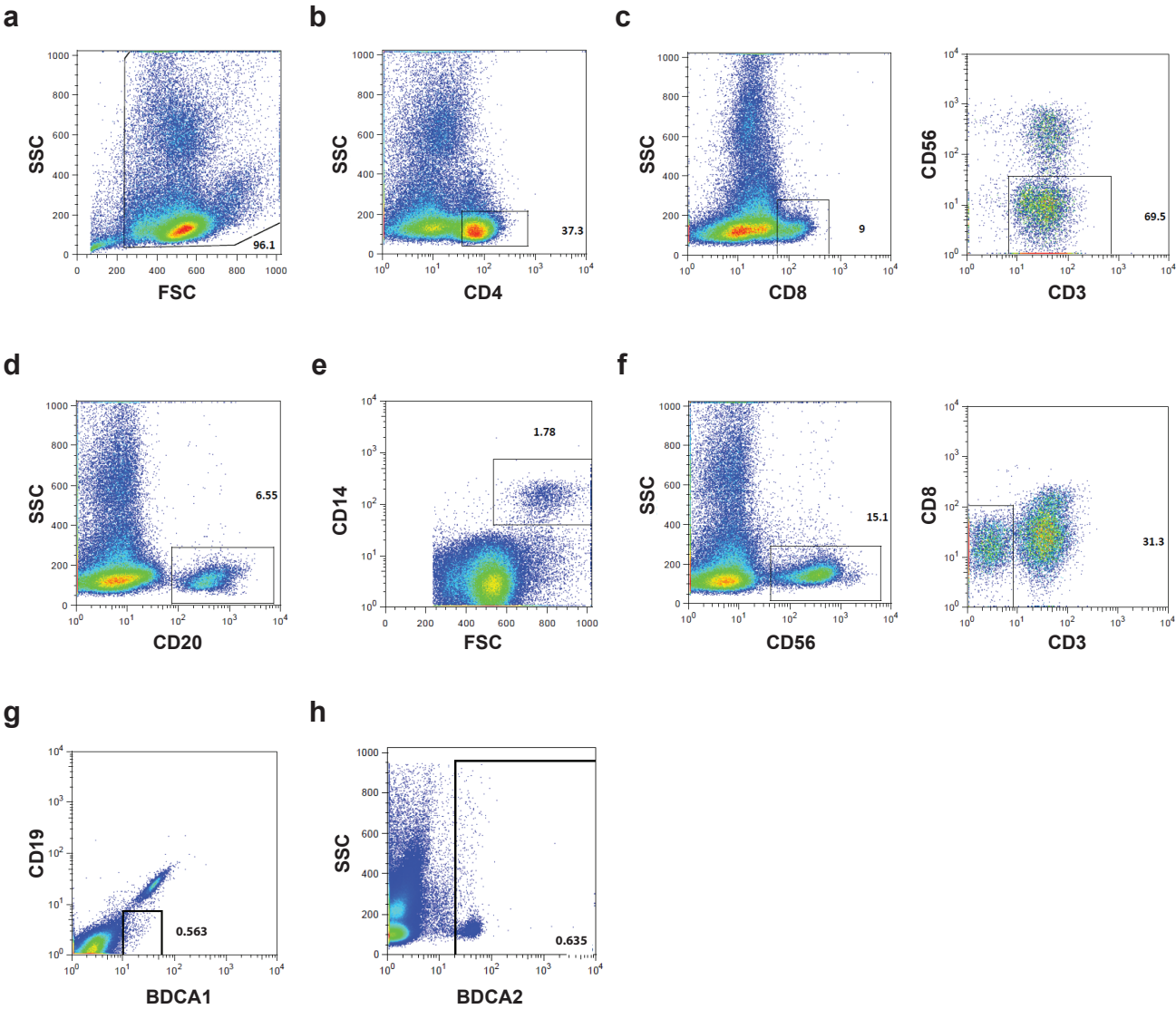

**Figure S2**

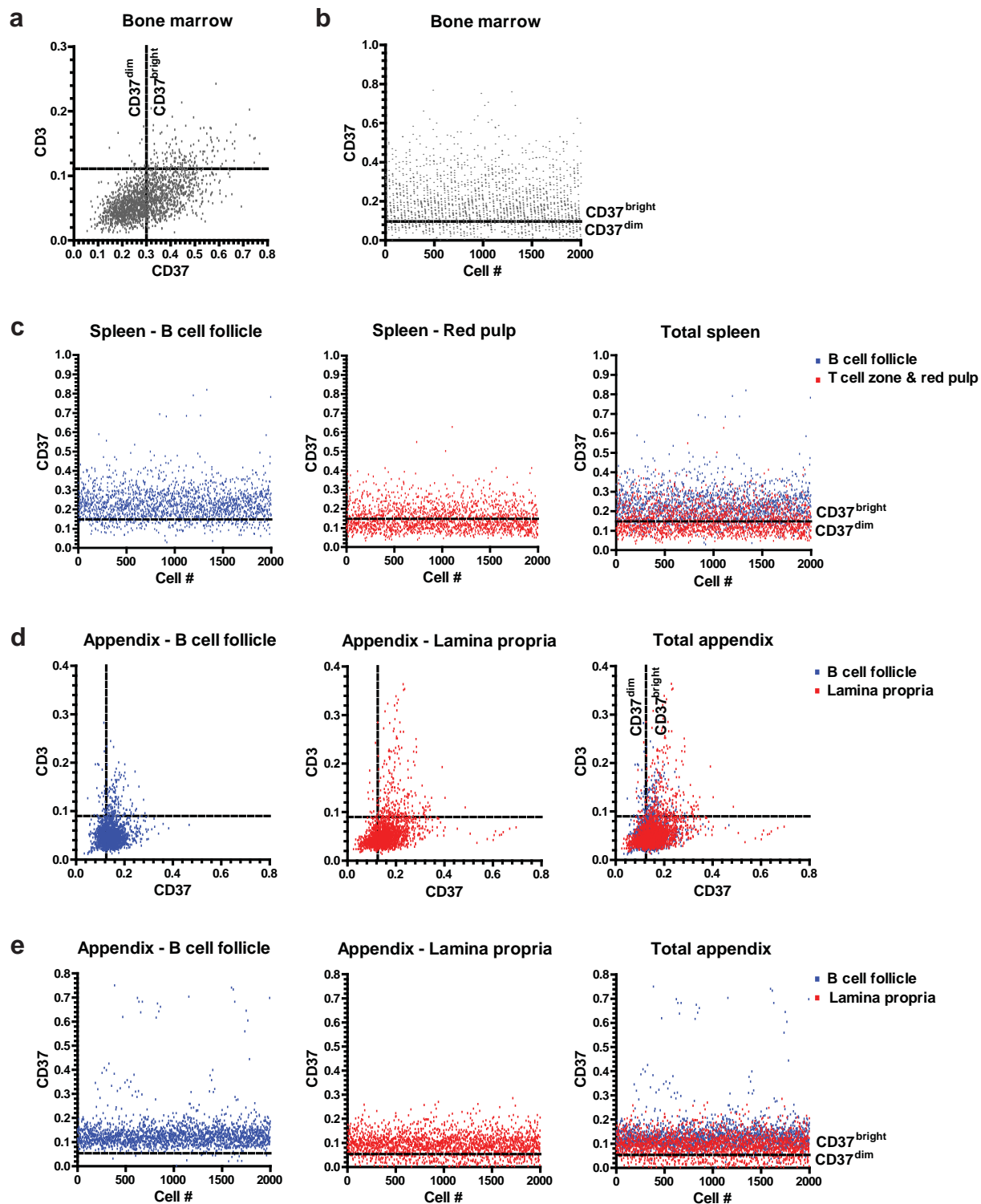

Figure S3

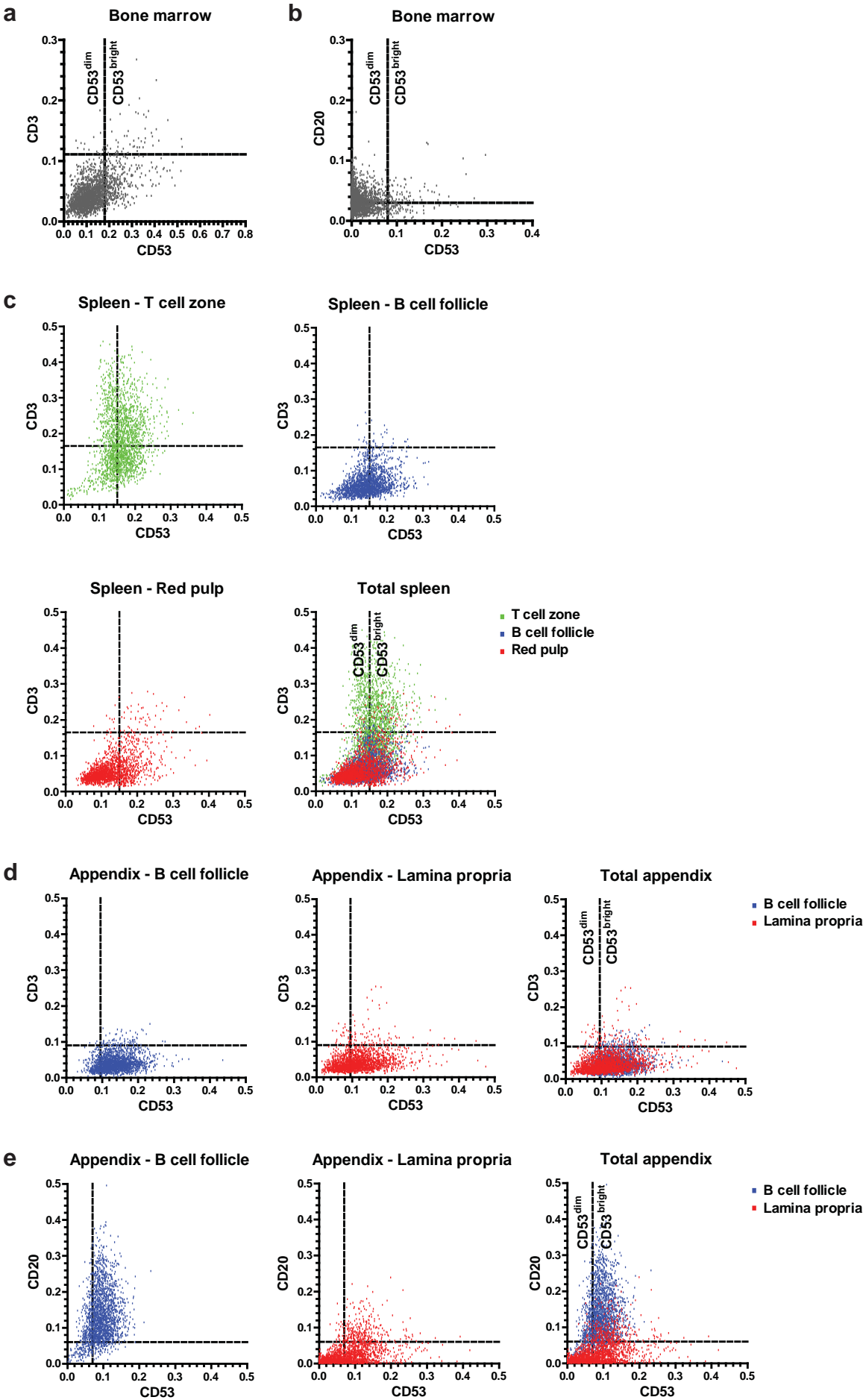

Supplement: Supplementary file 3 — Supplementary material 3 (PDF 2123 kb) [file 418_2015_1326_MOESM3_ESM.pdf]
